# Invasive Squamous Cell Carcinoma of the Scalp and Calvarium: A Multidisciplinary Approach

**Published:** 2016-11-08

**Authors:** Jennifer Stark, Silvio Podda, Karen Szymanski

**Affiliations:** Department of Plastic and Reconstructive Surgery, St. Joseph's Regional Medical Center, Paterson, NJ

**Keywords:** squamous cell carcinoma and chronic lymphocytic leukemia, skull base reconstruction, free flap reconstruction of the calvarium, microsurgical reconstruction, latissimus dorsi free flap

## Abstract

**Objective:** The correlation between immunosuppression-associated skin cancer and lymphoma has been well established. This includes squamous cell carcinoma and chronic lymphocytic leukemia. When a lesion requires excision, reconstruction can be challenging based on the depth and size of the tumor. We present a patient with chronic lymphocytic leukemia and invasive squamous cell carcinoma of the scalp that extended through the calvarium to the dura mater. His tumors were badly neglected for a long period of time and presented at an advanced stage. **Methods:** This type of reconstruction was performed utilizing a multidisciplinary approach. Our patient required calvarial reconstruction with titanium mesh, dural reconstruction, latissimus dorsi free flap, and an overlying skin graft. **Results:** The patient had appropriate resection of his tumor while maintaining flap viability. Postoperatively, he presented with excellent soft-tissue thickness and aesthetic result. **Conclusion:** We believe that this type of reconstruction was best, considering our patient had a significant scalp and calvarial defect at presentation. Using a latissimus dorsi free flap bestows a robust blood supply to help decrease infections and improve healing and circulation, especially in light of the need of further radiation therapy.

Squamous cell carcinoma (SCC) is the second most common skin cancer affecting 41 per 100,000 North Americans. Predilection occurs in sun-exposed areas, mainly the face, hands, and forearms. Other known risk factors include Fitzpatrick skin types I and II, carcinogen exposure, viral infections, radiation, chronic wounds, psoralen and ultraviolet A light exposure, and immunosuppression. The correlation between immunosuppression-associated skin cancer and lymphoma has been well established. This includes non-Hodgkin lymphoma (NHL) and chronic lymphocytic leukemia (CLL).^1^ The most common skin cancer types associated with NHL/CLL are melanoma, basal cell carcinoma, Merkel cell carcinoma, and SCC. Increased aggressiveness, higher recurrence rates, and increased regional metastasis have been correlated. Reconstruction of the scalp takes depth and size of the defect into account. Superficial defects less than 3 cm can be achieved with primary closure or local flaps. Adverse factors such as prior surgical intervention, radiotherapy, and scar formation can also affect the type of reconstruction performed. Larger defects with associated complicated scalp and calvarial defects have been shown to have the most favorable outcome with the use of free flaps. We present a patient with CLL and invasive SCC of the scalp that extended through the calvarium to the dura mater. Calvarial reconstruction with titanium mesh, dural reconstruction, and cutaneous reconstruction with a musculocutaneous free flap with an overlying skin graft are described.

## METHODS/CASE REPORT

**Figure 1:**
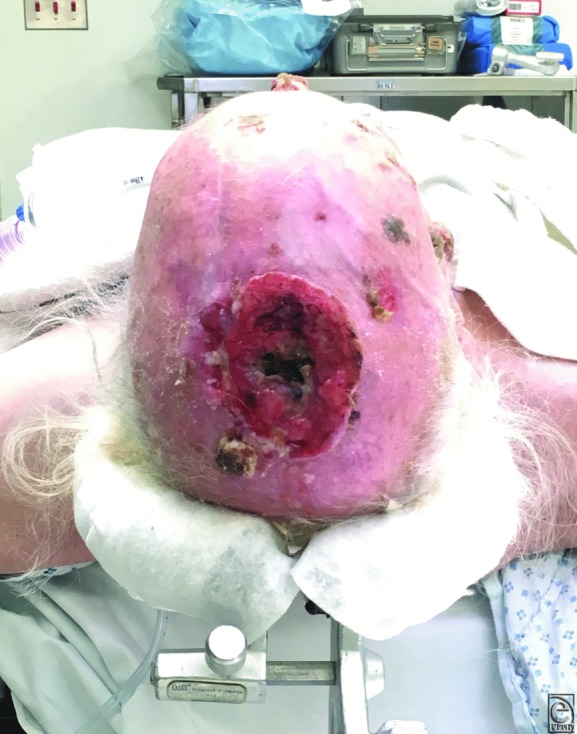
Scalp with invasion through to the calvarium/dura shown.

A 70-year-old man presented with a medical history significant for CLL and cutaneous squamous cell carcinoma (CSCC). The patient received chemotherapy in 2008 and has had a long-standing history of untreated SCC of his scalp and face for more than 10 years. He was initially scheduled for excision by a dermatologist but was delayed because of thrombocytopenia and then never followed up. Instead, he was applying a “cream” to the area with no relief. This lesion comprised a 6 × 6-cm, full-thickness defect that extended through the calvarium to the dura (Fig 1). The patient had experienced a brief period of headaches and left-sided numbness and tingling. Because of the complicated nature of this presentation, operative planning included a staged, multidisciplinary approach with neurosurgery, infectious disease, hematology/oncology, and microsurgical plastic surgery. Preoperative imaging and workup showed invasion of the bone and a bony defect, as well as potentially dural disease.

**Figure 2:**
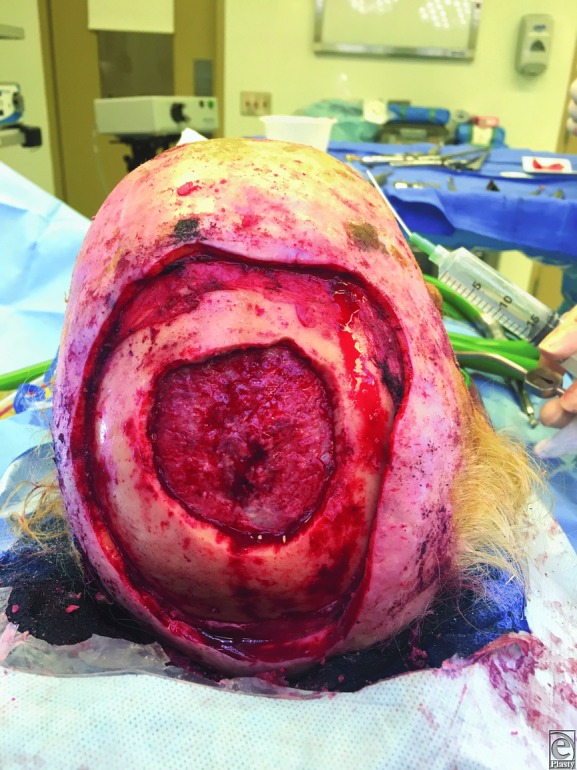
Excision of the tumor from scalp, calvarium, and dura.

**Figure 3:**
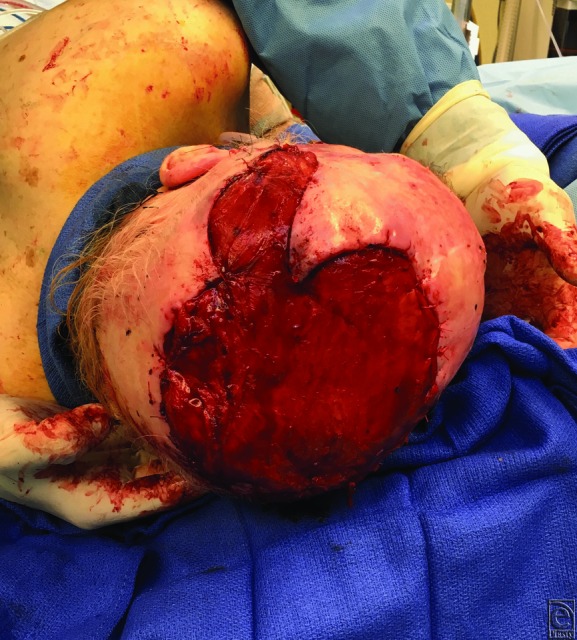
Latissimus dorsi flap contoured to the calvarium.

Initial surgery performed began with a 2-cm circumferential margin of the scalp (12 x 12-cm diameter defect), followed by craniectomy (6 x 6-cm diameter defect) and debridement of the dura. There was a partial-thickness dural defect in the center of the craniectomy (Fig 2). The second stage included titanium cranioplasty, additional dural surface debridement and Duragen (collagen matrix) placement, and a free latissimus dorsi flap. The superficial temporal artery and vein were anastomosed to the thoracodorsal artery and vein of the latissimus dorsi muscle using microsurgical principles (Fig 3). The third stage consisted of a split-thickness skin graft to cover the latissimus dorsi free flap.

## RESULTS

**Figure 4:**
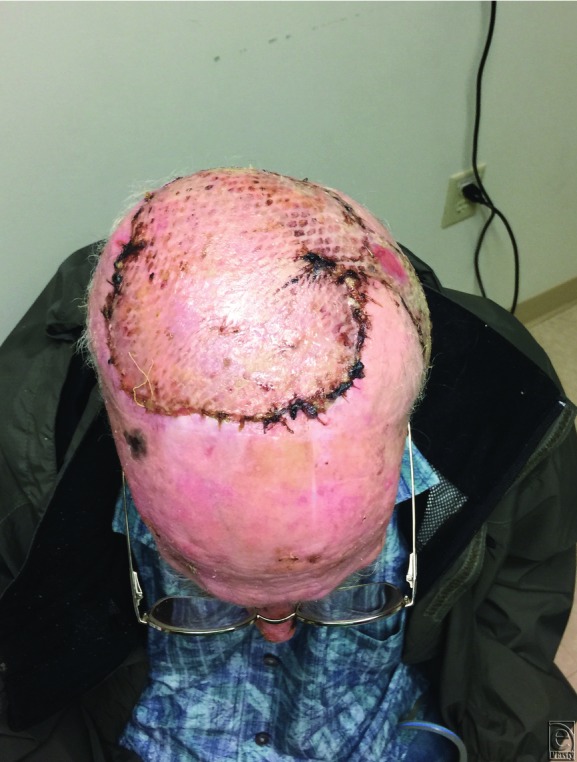
Postoperative result: 4 weeks after skin graft.

Pathology revealed appropriate negative margins after resection. The latissimus dorsi free flap provided the necessary coverage of the patient's scalp defect while restoring bony contour. His flap healed properly and remained closed without any signs of infection. As shown in Figure 4, the patient presents with excellent aesthetic results (4 weeks postoperatively) in our office. The patient had received postoperative radiation therapy and had no signs of necrosis or flap compromise.

## DISCUSSION

Management of high-risk SCC is complex. Patients who are immunosuppressed are at an increased risk of developing skin cancer. These tumors tend to present with a worse prognosis as described with our patient. Immunocompromised patients, especially those with CLL, experience an aggressive CSCC with higher recurrence and mortality. In these patients, tumors have a high rate of recurrence (19% at 5 years).^2^ It is also correlated with a 13% risk of metastatic disease.^2^ Cooperative management with a hematologist/oncologist is crucial.

The extent of dural involvement and invasion is an important prognostic indicator. In a retrospective review, Van Tuyl and Gussack^3^ demonstrated that patients who had dural involvement had a 22% survival rate after 3 years whereas those without dural involvement had a survival rate of 83%.

Reconstruction of such a large scalp defect is a challenging endeavor, best approached with a multidisciplinary team. Combined efforts from a neurosurgeon and a plastic surgeon can be necessary to allow safe tumor excision and adequate flap reconstruction of the defect.^4^

We believe that microsurgical reconstruction with a latissimus dorsi flap is a reliable option for providing stable coverage of these types of defects. It allows aggressive surgical resection of the tumor and complex reconstruction. It also brings a robust blood supply to help decrease infections and improve healing and circulation, especially in light of the need of further radiation therapy.
